# Emerging Concepts and Evidence in Telematics Novel Approaches or Treatments for Spasticity Management After Botulinum Injection

**DOI:** 10.3389/fresc.2021.720505

**Published:** 2021-09-06

**Authors:** Helena Bascuñana-Ambrós, Mª Josep Nadal-Castells, Eliot Ramírez-Mirabal, Marta Beranuy-Rodriguez, Alberto Pintor-Ojeda, Jean-Claude Perrot-González

**Affiliations:** Physical Medicine and Rehabilitation Department, Sant Pau University Hospital, Barcelona, Spain

**Keywords:** spasticity, telehealth, stretch, exercise, botulinum toxin

## Abstract

There is a strong recommendation for the use of intramuscular botulinum toxin in patients with persistent or progressive spasticity affecting one or more joints and who have an identifiable therapeutic target. After a botulinum toxin injection, a stretching intervention improves the results of the treatment, and it should be performed by patients and/or caregivers after being trained by a therapist. Adherence to this recommendation remains low once the therapist stops following the patient. The COVID-19 pandemic has increased the use of telemedicine with different approaches to treat patients. There has been an increased use of motivational applications, with virtual reality software and real-life videos, which provide a gaming experience that increases adherence. There are programs with synchronous telehealth exercises guided by physical therapists or software with sensor-based technology that shows the range of motion (ROM) and strength of the muscles of a particular joint. These new approaches to patient follow-up appear to increase adherence to exercise because they need to be “watched and controlled” is achieved.

## Introduction

Spasticity is involuntary muscle overactivity, which leads to damage to the central nervous system (brain and spinal cord). It is presented in a variety of ways depending on the size, location, and age of the lesion and may have several harmful secondary effects, such as pain, deformity, and impaired function (1, 2).

Spasticity management is challenging due to the diversity of patient presentation and goals or aims of treatment. It will normally include a combination of physical and pharmacological management, often using a variety of different approaches according to the needs of an individual patient (1, 2).

Local intramuscular injection of botulinum toxin (BoNT) is an established, well-tolerated treatment in the pharmacological management of focal spasticity. There is a strong body of Level I evidence about its effectiveness in the management of spasticity in upper and lower limbs (1, 2).

Clinicians who are experienced in the assessment and management of spasticity should be the ones to inject BoNT. It should not be used in isolation, but as part of a coordinated multidisciplinary approach, involving physical management and therapy, to achieve the desired effect (1, 2). The clinical pathway that involves BoNT multidisciplinary treatment before the COVID-19 pandemic implied a model of presential approach.

The COVID-19 pandemic has spread the use of telemedicine with different approaches to evaluate and treat patients (3). Telehealth has been available for many decades but the COVID-19 experience has resulted in heightened awareness of telehealth among health service providers, patients, and society overall. The need to use telemedicine solutions during the COVID-19 pandemic has raised the importance to consider what role telehealth will have post-pandemic (4).

There has been an increased use of motivational applications (apps) and physical training, with software, tutorials, virtual reality programs, and real-life videos that provide a gaming experience that increases adherence to treatments (5–10).

At the same time, the COVID-19 pandemic has evidenced the deficits in the remote control of our patients with spasticity. The suspension of most of the non-urgent activity in almost all of the rehabilitation departments around the world had led our patients without treatment during a period that ranges from a few months to almost a year.

The clinical pathway for the BoNT treatments needs the patient to actually “come to the hospital” for the injection procedure, but the control of the benefits of the injection according to the objectives defined between patient and physician is far to be optimal in most cases. Nowadays, we have the possibility of remote patient monitoring (RPM) to be able to monitor and manage patients in non-traditional healthcare settings. RPM uses digital technologies to collect health data from individuals in one location, such as a home of the patient, and electronically transmit the information to healthcare providers in a different location for assessment and recommendations (11–13).

Applications or games can guide users and their main caregivers to be able to provide and assist specific treatment for patients to improve the effectiveness of the intervention. To perform the correct stretching in the muscles treated with BoNT injection should be a telehealth solution to permit training and follow-up to the users. Therefore, patients should be able to obtain a more profitable result.

There are several designs of non-invasive digital technologies for Physical Medicine and Rehabilitation. Some non-invasive digital devices may be automated to capture and transmit health data without any action from the patient (i.e., biosensor or wearable devices); whereas, other technologies may require the patient to submit their health data through a secure Web site, smartphone, or personal digital assistant (PDA) (11–13).

We have searched to find those tools for our patients with spasticity to be able to use telemedicine solutions for their management after BoNT injection of their spastic muscles.

## Materials and Methods

A total of three databases (PUBMED, the Cochrane Library, and Web of Science) and Google Scholar were searched from January 1, 2015, to March 31, 2021, focusing on systematic reviews and original articles and gaming or exercise apps related to telematics treatments for spasticity.

The keywords used in the search were spasticity, telemedicine, telehealth, rehabilitation, stretch, BoNT, game, and apps or applications.

## Results

We have not found any apps consisting of games that specifically target the stretching exercise after the BoNT muscle injection for the patient with spasticity after our specific search.

The number of articles related individually to rehabilitation, telemedicine, and stretching is striking, without being able to obtain a favorable result for our search. We have not found any app or game that included patients with spasticity with postBoNT treatment.

The only app we have found related to the patient with spasticity is named Spasticity Assessor, and it is associated with the Tardieu scale. The Spasticity Assessor application has been designed to provide an accurate and easy-to-use method to assess spasticity of the lower extremities in a clinical setting. This application, based on the principles of the Modified Tardieu scale, allows evaluating the spasticity of the gastrocnemius, soleus, hamstring muscles (in hip flexion 40° and 90°) and quadriceps. It can provide an accurate joint range of motion (ROM) and speed of motion values that cannot be obtained with a traditional goniometer. The use of this system to measure movement speed and joint ROM has been validated with studies that consisted of comparing the concurrent validity of the Smartphone system with a three-dimensional movement analysis system of reference of criteria (3DMA) applied, and correct usage can be used to provide real-time information to ensure correct execution of treatment and as a valuable teaching resource for inexperienced students and clinicians.

## Discussion

We have not found any apps or games specific for patients with spasticity; we have found only one app for evaluation related to the Tardieu Scale. On the other hand, we have found apps that target the rehabilitation, control, follow-up, and monitoring of patients in other diagnoses, mostly, conditions with musculoskeletal involvement and associated with pain, as is the case of the tennis elbow (14) or golfer's elbow (15). For both pathologies, we have found these easy-to-use apps that incorporate health education, stretching programs, and diaries to record symptoms and/or progress. To date, the use of these specific applications currently lacks evidence from randomized controlled trials.

There are several stretch training apps not designed for specific medical diagnoses, but for the general population. A search in Google carried out on January 1, 2021, produced more than 11,800,000 apps related to stretch exercise but none for patients with spasticity.

The systematic review by Nussbaum et al. (5) showed that mobile apps may have positive benefits when they are used to deliver exercise or gait training intervention. The systematic review done by Zhou et al. (6) also supports the use of apps in survivors of stroke. The work by Salgueiro et al. (10) also showed that there are few apps used for patients affected by stroke to treat their sensoria-motor deficit with good acceptance. Neither of those systematic reviews included apps specific for spasticity.

There are apps for the more appropriate management of medication at home. It is estimated that 20–50% of patients do not take their medication correctly, and this leads to increase morbidity and inefficacy of pharmacological treatment. There are apps to assure treatment adherence to oral medications (7), and they have proved that is appropriate to manage medication at home.

There are some systematic reviews about how the apps can increase adherence to exercise in the general population, adults, and children that showed significant health improvement (8). Furthermore, there is modest evidence showing that, to date, apps are more effective in the short term (up to 3 months) (9).

The review done by Bonnechère et al. (16) concluded that the integration of commercial videogames into physical rehabilitation for various pathological conditions, such as stroke, cerebral palsy, Parkinson's disease, balance training, weight loss, and aging, showed that in most cases, the introduction of videogame training in physical rehabilitation offered similar results as conventional therapy. Therefore, videogames could be added as an adjunct treatment in rehabilitation for various pathologies to stimulate the motivation of the patients and/or to maintain rehabilitation benefits.

Why are there not any apps or games developed for patients with spasticity? Furthermore, why are other apps related to stretch for the general population not adapted for patients with spasticity? To develop them is mandatory to change the unwillingness of most clinicians to adopt telehealth (17). Why is it so difficult to adopt telehealth alternatives to usual care? Mainly, because it is complex (18), and it requires learning new methods of consulting and treating patients (19). In addition, it requires the clinician to accept them as effective, safe, and normal (17).

We should remark that we have not found any apps or games that focus on the patient with spasticity, and telerehabilitation *via* apps or games can be a solution because it eases access to patients, can increase the intensity of the rehabilitation process, and provides, in a cost/effective way, the continuity of care that the patient needs. In this article, we wanted to highlight the need for developing apps and games specifically for patients with spasticity, empowering patients and caregivers, and integrating telehealth into routine care.

## Author Contributions

All authors listed have made a substantial, direct and intellectual contribution to the work, and approved it for publication.

## Conflict of Interest

The authors declare that the research was conducted in the absence of any commercial or financial relationships that could be construed as a potential conflict of interest.

## Publisher's Note

All claims expressed in this article are solely those of the authors and do not necessarily represent those of their affiliated organizations, or those of the publisher, the editors and the reviewers. Any product that may be evaluated in this article, or claim that may be made by its manufacturer, is not guaranteed or endorsed by the publisher.
